# Hepcidin and Iron Homeostasis during Pregnancy

**DOI:** 10.3390/nu6083062

**Published:** 2014-08-04

**Authors:** Mary Dawn Koenig, Lisa Tussing-Humphreys, Jessica Day, Brooke Cadwell, Elizabeta Nemeth

**Affiliations:** 1 Department of Women, Children and Family Health Science, College of Nursing, University of Illinois at Chicago 845 S. Damen Ave., Room 814 (MC802), Chicago, IL 60612, USA; 2 Division of Health Promotion Research, Department of Medicine, University of Illinois at Chicago, Chicago, IL 60608, USA; E-Mail: ltussing@uic.edu; 3 University of Illinois Cancer Center, 1747W. Roosevelt Rd. #416, Chicago, IL, 60608, USA; 4 Sumter Family Health Center, 1278 N Lafayette Drive Sumter, SC 29150, USA; E-Mail: jday@sumterfhc.com; 5 School of Nursing, Yale University, 100 Church Street South, New Haven, CT 06519, USA; E-Mail: brooke.cadwell@yale.edu; 6 UCLA, Department of Medicine, Center for Iron Disorders, University of California Los Angeles, CHS 52-239, 10833 Le Conte Ave. Los Angeles, CA 90095-1690, USA; E-Mail: enemeth@mednet.ucla.edu

**Keywords:** hepcidin, pregnancy, iron regulation, inflammation

## Abstract

Hepcidin is the master regulator of systemic iron bioavailability in humans. This review examines primary research articles that assessed hepcidin during pregnancy and postpartum and report its relationship to maternal and infant iron status and birth outcomes; areas for future research are also discussed. A systematic search of the databases Medline and Cumulative Index to Nursing and Allied Health returned 16 primary research articles including 10 human and six animal studies. Collectively, the results indicate that hepcidin is lower during pregnancy than in a non-pregnant state, presumably to ensure greater iron bioavailability to the mother and fetus. Pregnant women with undetectable serum hepcidin transferred a greater quantity of maternally ingested iron to their fetus compared to women with detectable hepcidin, indicating that maternal hepcidin in part determines the iron bioavailability to the fetus. However, inflammatory states, including preeclampsia, malaria infection, and obesity were associated with higher hepcidin during pregnancy compared to healthy controls, suggesting that maternal and fetal iron bioavailability could be compromised in such conditions. Future studies should examine the relative contribution of maternal *versus* fetal hepcidin to the control of placental iron transfer as well as optimizing maternal and fetal iron bioavailability in pregnancies complicated by inflammation.

## 1. Introduction

Iron is essential for many metabolic processes, including oxygen transport and regulation of cell growth and differentiation [1,2]. Iron deficiency most prominently causes anemia, limiting oxygen delivery to cells, but iron deficiency can also cause dysfunction of the epithelia, muscle, and the nervous system [3]. On the other hand, excess amounts of iron in cells and tissues can result in tissue toxicity [1,4].

**Figure 1:**
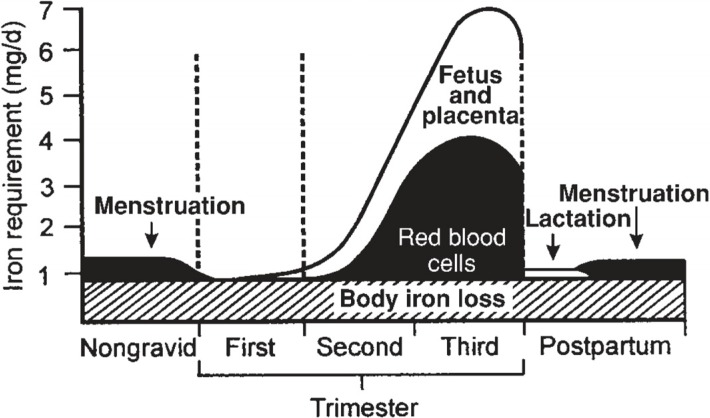
Estimated daily iron requirements during pregnancy in a 55-kg woman [5]. (with permission to publish photo from The American Journal of Clinical Nutrition).

Iron requirements increase nearly 10-fold during pregnancy from 0.8 mg/day in the first trimester to 7.5 mg/day in the third trimester (Figure 1) [5]. During pregnancy, iron is needed to support placental and fetal growth [6], sustain the increase in maternal red blood cell mass [7], and compensate for blood losses that will occur during delivery [8]. The Recommended Dietary Allowance (RDA) for iron increases from 18 mg/day for non-pregnant females to 27 mg/day during pregnancy [2]. Importantly, the RDA does not take into account varying bioavailability of iron among individuals. The median dietary iron intake among pregnant women is approximately 15 mg/day [2], an amount insufficient to support the increased demand during pregnancy. Therefore, many major health organizations recommend iron supplementation throughout pregnancy. The Centers for Disease Control and Prevention recommends routine low-dose iron supplementation (30 mg/day) for all pregnant women [9] and the Institute of Medicine also supports iron supplementation during pregnancy [2].

Iron is important for early placental development, which maintains pregnancy and provides nutrients and oxygen to the developing fetus [10,11,12,13,14]. Iron deficiency can adversely impact birth outcomes and result in preterm birth and low birth weight [15,16,17,18,19]. The mechanisms by which iron deficiency may affect birth outcomes are unknown, but the effects of hypoxia, oxidative stress, and increased risk of infection have been proposed as potential pathways [20]. Hypoxia, as a result of iron deficiency, could initiate a stress response, including the release of corticotropin-releasing hormone from the placenta and increased production of cortisol by the fetus, both of which are associated with increased risk of preterm birth [21,22]. Iron deficiency is associated with increased oxidative stress [23] and this could damage the placenta during early development [24,25,26]. Iron deficiency can also negatively impact the immune response [27] and thus increase the risk of infection during pregnancy.

**Figure 2:**
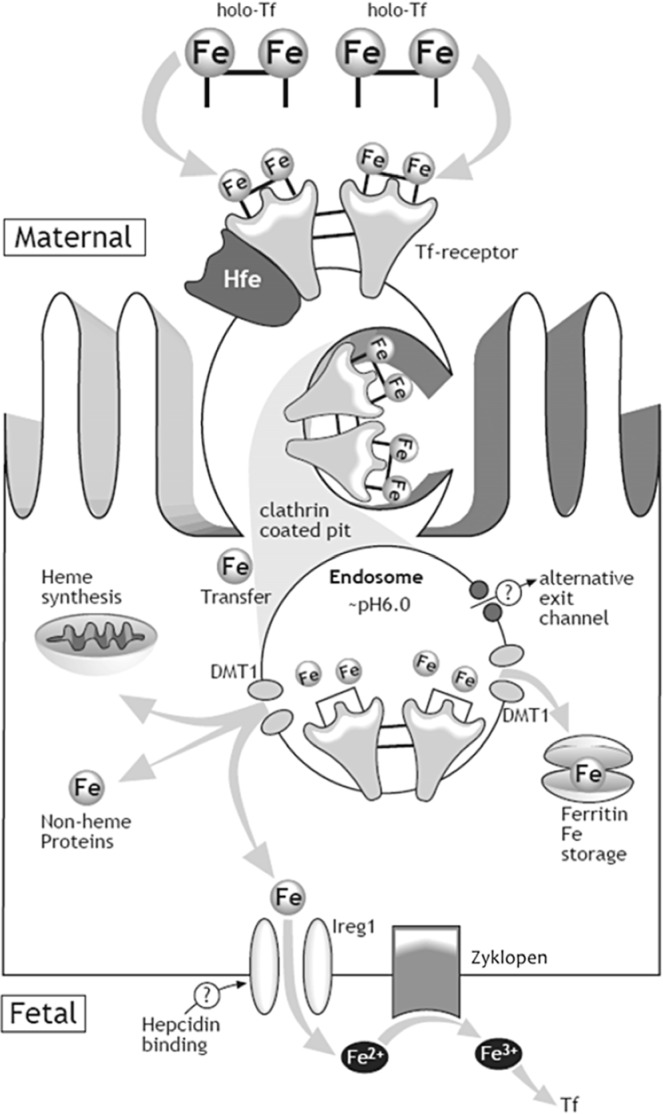
Mechanism of iron transfer across the placenta. The syncytiotrophoblast takes up iron-transferrin from maternal circulation via the transferrin receptors (TfR) cycle. After the holo-Tf/TfR complex is endocytosed, and iron is released from the complex in the acidifed endosome, iron is transported into the cytosol likely by the divalent metal transporter-1 (DMT-1). Iron is exported from the basolateral side of the synctiotrophoblast into fetal circulation by ferroportin (Ireg1). A ferroxidase (possibly zyklopen) oxidizes iron so it can be loaded onto fetal transferrin [28]. (with permission to publish photo from Nutrition Reviews).

Our current understanding of the mechanism by which iron is transferred from mother to fetus is depicted in Figure 2 [28]. Iron (Fe) is carried in the maternal blood bound to transferrin (Tf). The syncytiotrophoblast, the epithelium on placental villi that interfaces with maternal blood and participates in nutrient exchange, contains Tf receptors (TfR) on the surface facing maternal circulation. The binding of Fe-Tf to TfR depends on the pH levels, having a greater affinity at pH 7.4 [29]. After binding, the Fe-Tf/TfR complex is endocytosed, and the vesicle is acidified through activation of hydrogen pumps [30]. The lower pH allows the dissociation of iron from the maternal Tf. Once released, iron is actively transported out of the vesicle into the cytosol where it is used for cellular processes, stored in ferritin or exported into fetal circulation. The TfR and Tf then return to the maternal surface of syncytiotrophoblast where Tf is released into the maternal circulation and the cycle repeats [30]. Iron is exported from the basolateral side of the synctiotrophoblast into fetal circulation by ferroportin (Fpn) [30], the only know iron exporter in vertebrates. In fact, knockout of Fpn in mice causes embryonic lethality due to severe iron deficiency of the embryo [31]. Following iron export through Fpn, a ferroxidase (possibly zyklopen) [32] oxidizes iron so it can be loaded onto fetal transferrin.

**Figure 3:**
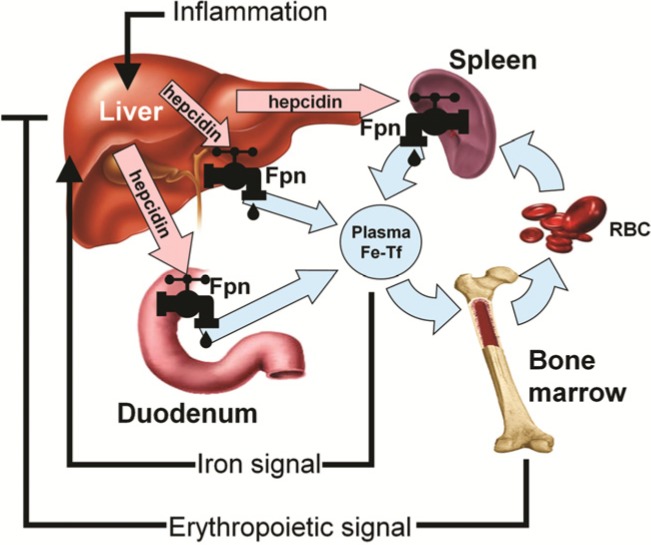
Hepcidin-ferroportin (Fpn) interaction determines the flow of iron into plasma. Hepcidin concentration is in turn regulated by iron, erythropoietic activity, and inflammation [40]. (with permission to publish photo from Blood: Journal of the American Society of Hematology).

In the last decade, there has been a revolution in our understanding of systemic iron homeostasis. Hepcidin is a peptide hormone that functions as both the homeostatic regulator of systemic iron metabolism and a mediator of host defense. Sensing of circulating iron and iron stores is thought to occur in the liver, which is the primary site of hepcidin production and secretion [33,34,35]. Hepcidin production can be assessed by measuring liver hepcidin mRNA levels (in animal models) or by measuring hepcidin peptide in the serum or plasma (in humans and mice) [36]. Circulating hepcidin is rapidly excreted through the kidneys (half-life in circulation is several minutes), and the peptide can also be measured in urine. Because hepcidin production is predominantly regulated at the transcriptional level, hepcidin mRNA and protein levels show high correlation. Hepcidin controls the efflux of iron into plasma by regulating Fpn (Figure 3). In addition to the placenta, Fpn is located on tissues that actively export iron including intestinal enterocytes, reticuloendothelial macrophages, and hepatocytes [33]. Hepcidin triggers Fpn degradation, reducing iron flux from these tissues thereby decreasing plasma iron concentrations and systemic iron bioavailability [34,37]. Hepcidin production by the liver is simultaneously regulated by circulating and stored iron, erythropoietic activity, and inflammation [36]. Therefore, at any time, hepcidin expression is determined by the interplay of these pathways and the relative strength of each of the individual signals [38]. When body iron levels are elevated or inflammation or infection is present, liver hepcidin production is increased resulting in diminished Fpn expression. Conversely, when body iron levels are depleted or anemia or hypoxia exists, hepcidin expression is reduced, allowing for increased dietary iron absorption and mobilization from body stores via active Fpn. Furthermore, hepcidin concentrations markedly influence iron absorption and can affect the efficacy of iron repletion via supplemental or dietary sources [39]. Therefore, given that iron accessibility is critical throughout pregnancy for both the mother and developing fetus, and hepcidin regulates systemic iron bioavailability, it is important to evaluate hepcidin concentrations in both uncomplicated and complicated pregnancies and throughout gestation.

The purpose of this review is to examine primary human and animal research studies that assessed hepcidin during pregnancy and postpartum, discuss relationship with maternal and fetal iron status, determine gaps in the literature, and suggest future areas for research.

## 2. Methods

The databases Pubmed and Cumulative Index to Nursing and Allied Health Literature (CINAHL) were searched with the terms: “pregnancy”, “hepcidin” and “iron”. Studies were considered for this review if hepcidin-25, the bioactive form of hepcidin, was measured in urine, serum or plasma, or if tissue-level gene expression (mRNA) was examined. Studies that measured pro-hepcidin were excluded because this metabolite is biologically inactive [41], does not correlate with serum hepcidin concentrations [42], nor does it demonstrate expected correlations with iron or inflammatory parameters [42,43]. Two other studies that used a commercially available hepcidin enzyme-linked immunosorbent assay (ELISA) were also excluded [44,45] because the assay was not clinically validated and does not correlate with mass spectrometric measurements [46]. There was no language restrictions included in the search, however, all the articles retrieved were written in English. The references for each primary research article were crosschecked to ensure that all studies were included.

Data extracted from the human studies included study design, sample size, maternal health status, presence of obstetrical complications, gestational age, study location, maternal age, body mass index (BMI), race/ethnicity, inclusion and exclusion criteria, biological sample type, timing of specimen collection, method used to measure hepcidin, maternal and fetal hepcidin concentrations, and associations between hepcidin with clinical and biochemical parameters. Data extracted from the animal studies included study design, sample size, animal type, experimental groups, gestational age, diet, biological sample type, timing of specimen collection, and assay used to measure hepcidin, and maternal and fetal hepcidin concentrations.

## 3. Results and Discussion

Hepcidin is the systemic regulator of iron metabolism, and iron availability is critical during pregnancy for both mother and fetus. This review reveals a significant paucity of studies assessing hepcidin during pregnancy. Ten human [47,48,49,50,51,52,53,54,55,56] and six animal studies [57,58,59,60,61,62] met the inclusion criteria with findings reported in Table 1.

**Table 1:** Studies included in review of hepcidin values in pregnancy.

Author	Design	Sample	Hepcidin	Main Study Findings
Human Studies				
Finkenstedt *et al.* (2012) [54]	Longitudinal, prospective	*n* = 42 Health status: 38 healthy and 4 with complications (preeclampsia: *n* = 1, GDM: *n* = 1, C/S: *n* = 1, preeclampsia and GDM: *n* = 1). Location: NDR Maternal age (years): 26.4 (range 17–40) BMI (kg/m^2^): NDR Parity: primigravida: *n* = 7, previous pregnancy mean = 1.6 Race/Ethnicity: NDR Inclusion/Exclusion criteria: NDR	Sample: Maternal serum Timing: 1st and 3rd trimester (*n* = 42); 2nd trimester (*n* = 12) Method: SELDI time-of-flight mass spectrometry [63] Hepcidin reference range: average for healthy women 50 ng/mL (range < 10–200 ng/mL)	Maternal serum hepcidin: 1st trimester: median = 16 ng/mL (4–97); 2nd trimester: median = 11 ng/mL (6–36); 3rd trimester: median = 9.5 ng/mL (1–43), *p* < 0.001 Correlations of maternal hepcidin with iron and inflammatory parameters: Serum iron (*r* = 0.391, *p* < 0.001), ferritin (*r* = 0.573, *p* < 0.001), Tsat (*r* = 0.457; *p* < 0.001), sHJV (*r* = −0.231; *p* = 0.025), EPO (*r* = −0.308; *p* = 0.002).
van Santen *et al.* (2013) [55]	Longitudinal, prospective	*n* = 31 Health status: healthy except for complications in 3 (10%) Location: The Netherlands Maternal age (years): 33.1 ± 5.4 BMI (kg/m^2^): NDR Parity 0 = 13 (42%), Parity 1 = 11 (36%), Parity ≥ 2 = 7 (23%) Race/Ethnicity: Northern European (*n* = 29) or Southeast Asian (*n* = 2) Inclusion criteria: Normal hematologic blood count, renal function and liver enzymes at first visit	Sample: Maternal serum Timing: 9–15, 19–25 and 29–35 weeks gestation; within 24 h postpartum; 6 weeks post-delivery Method: combination of weak cation exchange chromatography and time-of-flight mass spectrometry [64] lower limit of detection: 0.25 nmol/L Hepcidin reference range: median 2.0 nmol/L (range < 0.5–12.3 nmol/L)	Maternal serum hepcidin: 15–19 weeks gestation: 1.85 nmol/L (1.10–4.10); 20–25 weeks gestation: 0.25 nmol/L (0.25–1.20); 29–35 weeks gestation: 0.25 (0.25–0.25, undetectable); 24 h postpartum: 3.0 nmol/L (0.66–9.22); 6 weeks post-delivery: 1.35 nmol/L (0.73–2.40) Correlations of maternal hepcidin with iron and inflammatory parameters: Serum ferritin (*r*^2^ = 0.516, *p* < 0.0001), serum iron (*r*^2^ = 0.1, *p* = ns), Hgb (*r*^2^ = 0.275, *p* = 0.015), TIBC (*r*^2^ = −0.483, *p* < 0.0001), sTfR (*r*^2^ = −0.293, *p* = 0.008), sTfR-index (*r*^2^ = −0.452, *p* < 0.001), and Tsat (*r*^2^ = 0.243, *p* = 0.029), CRP (*r*^2^ = 0.322, *p* = ns)
Dao *et al.* (2013) [56]	Longitudinal, prospective	*n* = 30 Health status: (*n* = 15 Lean; *n* = 15 Obese) GDM (Obese, *n* = 1; Lean, *n* = 1) OB complication: C/S (Obese, *n* = 7; Lean, *n* = 4) Location: Boston, MA, USA Maternal age (years): Obese: 30.0 ± 3.9; lean 32.1 ± 5.8 BMI (kg/m^2^): Obese = 38.6 ± 7.0; Lean = 22.8 ± 1.5 Parity: NDR Race/Ethnicity: Obese: Caucasian *n* = 6; AA *n* = 6; Hispanic *n* = 2; Asian *n* = 1; Lean: Caucasian *n* = 9; AA *n* = 0; Hispanic *n* = 5; Asian *n* = 1 Exclusion Criteria: type 2 diabetes, preeclampsia, autoimmune disease, acute infection, premature membrane rupture and chorioamnionitis	Sample: Maternal serum and cord blood Timing: 24–28 weeks gestation (maternal sample); childbirth (cord blood) Method: ELISA (Bachem Group, Torrance, CA, USA) Hepcidin reference range: NDR	Maternal serum hepcidin: (24–28 weeks gestation): Obese: 13.5 ± 9.0 ng/mL; Lean: 5.1 ± 2.7 ng/mL (*p* < 0.01). Correlated with maternal BMI: *r* = 0.4, *p* = 0.04 Maternal inflammatory status (24–28 weeks gestation): CRP: Obese: 14.3 (IQR: 11.5) mg/L; Lean: 5.0 (IQR: 4.4) mg/L (*p* < 0.01) CRP: Obese: 14.3 (IQR: 11.5) mg/L; Lean: 5.0 (IQR: 4.4) mg/L (*p* < 0.01) Maternal iron status (24–28 weeks gestation) (extrapolated from Figure 1): Serum iron: Obese: 60 μg/dL; Lean: 78 μg/dL Tsat: Obese: 15% Lean: 19% Cord blood hepcidin (Childbirth) (extrapolated from Figure 1): Obese: 125 ng/mL; Lean: 120 ng/mL Cord blood iron status parameters Serum iron: Obese: 97.3 ± 29.9 μg/dL; Lean: 147.7 ± 21.7 μg/dL (*p* < 0.01); Tsat: Obese: 39.6%; Lean: 63.5% (*p* = 0.01) Correlations of maternal parameters and cord blood iron status markers: Maternal BMI and cord blood iron: *r* = −0.8, *p* = 0.002; Maternal BMI and cord blood Tsat: *r* = −0.7, *p* = 0.009; Log maternal hepcidin and cord blood iron: *r* = −0.6, *p* = 0.02; Log maternal hepcidin and cord blood Tsat: *r* = −0.6, *p* = 0.02
Gyarmati *et al.* (2011) [53]	Longitudinal, prospective	*n* = 38 Health status: healthy; elective C/S *n* = 13 Location: Hungary Maternal age: NDR BMI: NDR Parity: NDR Race/ethnicity: NDR Exclusion criteria: presence of infection, multiple birth, and any known OB complication	Sample: Maternal serum Timing: median 40 (39–41) weeks gestation; at first contraction for vaginal delivery, or before anesthesia for elective C/S, and then 3 days PP for all Method: mass spectrometry [50] Hepcidin reference range: NDR	Maternal serum hepcidin: Vaginal: before birth 2.52 ng/mL (2.07–3.1); 3 days PP 7.36 ng/mL(6.34–8.91) (*p* < 0.001); Elective caesarian delivery: before birth 2.83 ng/mL (2.53–3.87); 3 days PP 17.5 ng/mL (13.5–18.9) (*p* < 0.001) Correlations of maternal hepcidin with iron and inflammatory parameters: NDR; no significant correlation with iron, ferritin and IL-6.
van Santen *et al.* (2011) [51]	Cross-sectional, retrospective	*n* = 69 Health status: stratified by group: no placental malaria and no anemia (*n* = 21) no placental malaria and anemia (*n* = 18) placental malaria and no anemia (*n* = 16) placental malaria and anemia (*n* = 14). Location: Gabon Maternal age (years): range 16–19 BMI (kg/m^2^): NDR Parity: primigravid Race/Ethnicity: NDR Exclusion criteria: Primigravid without peripheral parasitemia, live singleton birth, no signs or symptoms of systemic infection	Sample: Maternal plasma and cord blood Timing: childbirth Method: combination of weak cation-exchange chromatography and time-of-flight mass spectrometry Hepcidin reference range: 4.2 nmol/L (0.5–13.9) nmol/L [65]	Maternal plasma hepcidin (Childbirth): group 1 = 4.2 (nmol/L) (1.6–8.0) group 2 = 2.8 (nmol/L) (0.5–13.1) group 3 = 4.2 (nmol/L) (2.0–8.1) group 4 = 3.2 (nmol/L) (0.9–5.0) *p* = 0.70 Cord blood hepcidin (Childbirth): group 1 = 4.5 (nmol/L) (2.8–6.8) group 2 = 5.5 (nmol/L) (2.4–9.5) group 3 = 3.7 (nmol/L) (3.0–6.9) group 4 = 4.4 (nmol/L) (1.8–6.5) *p* = 0.33 Correlations of maternal hepcidin with iron and inflammatory parameters: NDR
Young *et al.* (2012) [52]	Correlational, cross-sectional, prospective	*n* = 19 Health status: healthy Location: Rochester, NY, USA Maternal age (years): mean = 19.0 ± 2.9 (range 16–32) BMI (kg/m^2^): pre-pregnancy 24.7 [7.0] (20.7–43.6); delivery BMI: 30.4 [7.1] (24.4–47.5) Parity: mean = 0 ± 0.9 (0–3) Race/Ethnicity: African American 53%, Caucasian 47% Inclusion criteria: Healthy, nonsmoking, uncomplicated pregnancy Exclusion criteria: gestational diabetes, underlying malabsorption, or medical problems that affect Fe homeostasis	Sample: Maternal serum and cord blood Timing: mean = 39.9 ± 1.6 weeks (36–41.6) gestation; childbirth (maternal sample collected at admission) Method: ELISA Hepcidin reference range: median = 65 ng/mL (5%–95% range 17 to 286) [66]	Maternal serum hepcidin (Childbirth): Median [SD] = 9.30 μg/L [50.1] Cord blood hepcidin (Childbirth): Median [SD] = 61.7 μg/L [77.0], *p* < 0.05 Correlations of maternal hepcidin with iron and inflammatory parameters: ferritin (μg/L) (*r*^2^ = 0.59, *p* = 0.0001), TBI (mg/kg) (*r*^2^ = 0.59, *p* = 0.0001), Hgb (g/L) (*r*^2^ = 0.31, *p* = 0.01) and TfR (mg/L) (*r*^2^ = 0.31, *p* = 0.01)] Correlations of cord blood hepcidin with neonatal iron and inflammatory parameters: ferritin (*r*^2^ = 0.60, *p* < 0.0001) and TBI (*r*^2^ = 0.60, *p* = 0.0001) Maternal serum hepcidin did not significantly correlate with neonatal serum hepcidin. Pregnant women with undetectable levels of serum hepcidin transferred a greater quantity of the maternally ingested ^57^Fe-nonheme or 58Fe-heme iron to their fetus compared to women with detectable levels of serum hepcidin (*p* = 0.003 and 0.002)
Rehu *et al.* (2010) [48]	Cross-sectional, prospective, correlational	*n* = 191 Health status: vaginal delivery: *n* = 147; elective C/S: *n* = 24; emergency C/S: *n* = 20; gestational diabetes mellitus: *n* = 23 gestational hypertension: *n* = 6; preeclampsia: *n* = 5; liver dysfunction: *n* = 2; three groups: 1. iron-restricted erythropoiesis; 2. sufficient iron for erythropoiesis, but low iron stores; 3. normal iron stores and sufficient iron for erythropoiesis Location: Finland Maternal age (years): median = 28 (range 17–41) BMI (kg/m^2^): NDR Parity: NDR Race/Ethnicity: NDR Inclusion/Exclusion criteria: NDR	Sample: Maternal serum and cord blood Timing: median = 40 + 1 weeks (37–42) gestation; selected at time of delivery <24 h before delivery Method: ELISA Hepcidin reference range: median 65 ng/mL 5%–95% range 17 to 286 [66]	Maternal serum hepcidin (Childbirth): Overall geometric mean = 12.4 ng/mL (95% CI = 10.5–14.6); vaginal geometric mean = 13.6 (95% CI = 11.0–17.0); emergency CS geometric mean = 15.9 (95% CI = 8.8–28.7); elective CS geometric mean = 5.5 (95% CI = 3.2–9.5), significantly lower than vaginal or emergency CS; iron-restricted erythropoiesis = 3.8 ng/mL (1.5–9.6); low iron stores = 6.7 ng/mL (4.2–10.6); normal iron stores = 15.2 ng/mL (11.1–20.7), *p* = 0.002 Correlations of maternal hepcidin with iron and inflammatory parameters: CRP (*r* = 0.285, *p* < 0.001) Cord blood hepcidin (Childbirth): geometric mean = 71.6 ng/mL (95% CI = 60.8–84.4), *p* < 0.001; None of the maternal measurements were associated with the cord blood hepcidin
Toldi *et al.* (2010) [50]	Cross-sectional, descriptive	*n* = 67 Health status: 30 preeclamptic and 37 healthy Location: Hungary Maternal age (years): median = 30 BMI (kg/m^2^): NDR Parity: NDR Race/Ethnicity: NDR Inclusion/Exclusion criteria: not reported; routine perinatal oral iron supplementation: all participants.	Sample: Maternal plasma Timing: 24–40 weeks gestation; preeclamptic median = 36.5 (24–40) weeks gestation; healthy median = 36 (28–39) weeks gestation Method: mass spectrometry (LC-MS/MS) [36]	Maternal plasma hepcidin 24–40 weeks gestation:Preeclampsia: 5.68 ng/mL (0.72–9.25) Healthy: 3.74 ng/mL (0.73–8.14) (*p* = 0.003) Correlations of maternal hepcidin with iron and inflammatory parameters: NDR
Schulze *et al.* (2008) [49]	Correlational, cross sectional prospective.	*n* = 190 Health status: purposive sampling of women with iron deficiency Location: Bangladesh Maternal age: mean = 21.9 (17.5–25) years BMI (kg/m^2^): NDR Parity: 48% primigravida; 52% multigravida Race/Ethnicity: NDR Inclusion/Exclusion criteria: not reported	Sample: Maternal urine Timing: mean (SD) = 12 (8–14) weeks gestation; following pregnancy confirmation Method: surface-enhanced laser desorption/ionization time-of-flight mass spectrometry Hepcidin reference range: 0.09–2.97 [67]	Maternal urine hepcidin 8-14 weeks gestation: median = 2.35 (intensity/mmol creatinine) (0.51–7.22) Correlations of maternal hepcidin with iron and inflammatory parameters: ferritin (μg/L) (*r* = 0.33, *p* < 0.001) & TfR index (TfR/log ferritin) (*r* = −0.22, *p* = 0.007); no correlation with soluble TfR, hemoglobin, EPO; log AGP (mg/dL) (*r* = 0.20, *p* = 0.01); no correlation CRP
Howard *et al.* (2007) [47]	Correlational, cross-sectional, prospective	*n* = 31 Health status: + Plasmodium Falciparum malaria Location: Ghana Maternal age (years): mean = 24.4 BMI (kg/m^2^): NDR Parity: NDR Race/Ethnicity: NDR Inclusion criteria: diagnosed with P. Falciparum malaria, not admitted for transfusion, had a hemoglobin level >50 g/L, and no cerebral malaria	Sample: Maternal urine Timing: NDR Method: time-of-flight mass spectrometry Hepcidin reference range: 0.09–2.97 intensity/mmol creatinine [67]	Maternal urine hepcidin: mean = 0.7 (intensity/mmolcreatinine) (0.2, 5.9) Correlations of maternal hepcidin with iron and inflammatory parameters: not associated with hemoglobin or anemia, but 90% of women were anemic; log parasitemia positively associated (β = 0.218; CI = 0.043–0.392, *p* = 0.016)
Animal Studies				
Cornock *et al.* (2013) [61]	Prospective, cross-sectional	*n* = 40 Animal: virgin Wistar and Rowett Hooded Lister rats Age: NDR Diet: Dams fed either Control iron (50 mg/kg) Low iron (7.5 mg/kg)	Sample: maternal and fetal liver Timing: day 21of gestation, placentas and livers dissected and frozen Method: mRNA by real-time RT-PCR	Maternal liver hepcidin: mRNA hepcidin expression decreased in rats fed low iron diet (*p* < 0.001), no difference by strain Fetal liver hepcidin: mRNA hepcidin expression higher in Wistar compared to Rowett Hooded Lister (43%, *p* < 0.001), and was decreased by exposure to low iron diet in both strains (61%, *p* < 0.001)
Sozo *et al.* (2013) [62]		*n* = 13 Animal: Border-Leicester × Merino ewes Groups: Ethanol 0.75 g/kg (*n* = 8), daily IV administration on day 95–133 of gestation (3rd trimester) Saline (*n* = 7)	Sample: fetal liver Timing: gestational day 134 (term is ~day 145) Method: mRNA by real-time RT-PCR	Fetal liver hepcidin: Gene expression lower in ethanol-exposed liver (0.2 ± 0.1) compared to control (1 ± 0.2), *p* = 0.011
Neves *et al.* (2010) [58]	Prospective, cross sectional	*n* = 13 Animal: mice Groups: C57BL/6 (B6), *n* = 6 Hfe−/− knockout, *n* = 7 Age: one year old nulliparous and pluriparous (mean = 29 weaned pups per female) Diet: fed standard diet with iron content of 312 mg/kg	Sample: female liver Timing: 1 year old. For pluriparous group, the sample was collected on average 11 weeks after last delivery Method: hepcidin 1 and 2 mRNA by real-time RT-PCR	Hepcidin 1 and 2: lower expression of hepatic hepcidin mRNA in pluriparous mice (of both genotypes) compared to nulliparous (*p* < 0.01)
Gambling *et al.* (2009) [59]	Experimental, correlational, prospective	*n* = 104 Animal: virgin Rowett Hooded Lister Rats Groups: control group supplemented in 1st half of pregnancy, iron deficient in the 2nd half iron deficient diet for 1st half, supplemented 2nd half deficient diet throughout	Sample: maternal and fetal liver Timing: fetuses delivered by C/S on day 21.5 of gestation, Method: real time quantitative PCR from total RNA from liver	Maternal liver hepcidin: mRNA hepcidin expression decreased in iron deficient group (*p* < 0.001), hepcidin restored to near control levels by iron supplementation in the 2nd half but not 1st half of pregnancy (*p* < 0.05) Fetal liver hepcidin: Low in fetuses from iron deficient group (*p* < 0.001); fetal liver iron and maternal liver hepcidin (*r*^2^ = 0.59, *p* < 0.001); fetal liver iron and hepcidin expression in fetal liver (*r*^2^ = 0.47, *p* < 0.001)
Millard *et al.* (2004) [60]	Cross-sectional, Prospective	*n* = 4 per time point Animal: Sprague-Dawley rats Age: mated at 10–12 weeks, those not pregnant used as control Diet: All fed standard rodent pellet diet (370 mg iron/kg)	Sample: maternal liver Timing: 5 time points: 9, 15, 18, and 21 days gestation, and 24–48 h PP Method: hepatic samples by ribonuclease protection assay	Maternal hepatic hepcidin mRNA: Progressive decline through gestation down to 1.9% of the non-pregnant level on day 21 (*p* < 0.05); Postpartum levels normalized

Note: NDR = no data reported; BMI = body mass index; sTfR = soluble transferrin receptor; C/S = cesarean section; GDM = gestational diabetes mellitus; IQR = interquartile range; PP = postpartum; CI = confidence interval; TIBC = total iron binding capacity; IL-6 = Interleukin-6; Hgb = hemoglobin level; CRP = C-reactive protein; EPO = erythropoietin; TfR index = transferring receptor index (TfR/log ferritin); AGP = alpha-1 acid glycoprotein; TBI = total body Fe; TfR = transferring receptor; HFE = Human hemochromatosis protein; sHJV = soluble hemojuvelin; AGP = α_1_-acid glycoprotein; Tsat = transferrin saturation; ns = not significant.

Six of the ten human studies utilized a cross-sectional study design [47,48,49,50,51,52] and four were longitudinal [53,55,56,68]. Sample size for the human studies ranged from 19 [52] to 191 subjects [48]. Human studies examined hepcidin values at 8 to 15 weeks gestation [49,55], 19 to 25 weeks [55], 24 to 28 weeks gestation [56], 24 to 40 weeks gestation [50,55], at childbirth, [51,53], 24 h postpartum, [55] three days postpartum [53], and six weeks postpartum [55]. Only three studies examined hepcidin longitudinally across all three trimesters [55,68]. Cord blood hepcidin, obtained at delivery, was measured in four of the human studies [48,51,52,56]. Hepcidin was assessed in plasma [50,51], serum [48,52,53,55,56,68], and urine [47,49]. Seven of ten human studies analyzed hepcidin using mass spectrometry [47,49,50,51,53,55,68] and three studies used ELISAs [48,52,56]. Unfortunately, hepcidin values from the human studies cannot be directly compared to each other because hepcidin assays have not been standardized, and different methods yield different absolute values and normal ranges, although they correlate strongly [65]. It is unknown what magnitude of change in hepcidin value is biologically important during pregnancy. More research in larger populations is needed to determine the reference ranges for hepcidin during pregnancy.

The six animal studies (performed on ewes, rats, and mice) were longitudinal. In rodents, sample collection ranged from 9 day to 21.5 days of gestation, and 24–48 h post-delivery [59,60]. In ewes, the samples were collected during the third trimester. Five of the six animal studies measured hepatic hepcidin mRNA expression.

### 3.1. Maternal Hepcidin Levels through Pregnancy

Hepcidin in pregnant women is lower than in nonpregnant healthy women and hepcidin levels decrease as pregnancy progresses, with the lowest hepcidin levels observed in the third trimester (longitudinal studies, and those assessing the third trimester) [53,54,55,56]. The increased need for fetal iron in the third trimester may be responsible for the decrease in maternal hepcidin observed, as fetal demand for iron is greatest in the third trimester [69]. The signals that suppress maternal hepcidin during pregnancy are unknown. Pregnancy-specific regulators of hepcidin production may exist, or hepcidin may be suppressed in response to decreasing maternal iron levels during pregnancy.

Similarly, in animal studies liver hepcidin mRNA expression decreased throughout pregnancy. By day 21 in rodents (equivalent to the third trimester) hepcidin mRNA expression was 1.9% of non-pregnant values. However, hepcidin expression returned to pre-pregnancy concentrations within 24 to 48 h postpartum (*p* < 0.05) [60], possibly because of the stress response associated with delivery and/or the loss of unknown inhibitory signals produced during pregnancy. Rats fed a low iron diet had lower levels of both maternal and fetal mRNA hepcidin expression compared to the control group [61]. This is likely a result of hepcidin suppression in iron deficient states, allowing for more bioavailable iron to be used by the system. So far, animal and human studies observed similar decreases in hepcidin during pregnancy. In general, the hepcidin regulatory circuitries have been shown to be very similar between rodents and humans, but it remains to be determined if any pregnancy-related differences may exist.

### 3.2. Maternal Hepcidin and Iron Status

Maternal hepcidin concentrations were significantly correlated with indicators of maternal iron status [48,49,55]. During the first trimester of pregnancy, serum and urinary hepcidin were positively correlated with ferritin and negatively correlated with serum transferrin receptor (sTfR) index, a sensitive indicator of iron deficiency [49]. Similarly, throughout the gestational period, serum hepcidin correlated positively with ferritin and transferrin saturation and negatively with sTfR, and hemoglobin concentration [55]. This suggests that hepcidin regulation by iron and erythropoiesis is preserved in pregnancy [66]. Even at delivery (samples collected at admission), plasma hepcidin was associated with maternal hemoglobin, ferritin, total iron, and sTfR [52], and was found to be lowest in pregnant women with the poorest iron status [48]. However, one to three days postpartum, serum hepcidin levels increased, and did not correlate with serum iron or ferritin, likely due to the transient effect of the stress of delivery [51,53,55].

Hepcidin was reduced in women with low circulating iron and elevated erythropoiesis [48]. True iron deficiency anemia is defined by both lower ferritin values (<30 ng/mL) and hemoglobin (<12 g/dL) [70]. Further research with larger sample sizes and longitudinal designs are needed to determine how hepcidin changes throughout each trimester of pregnancy in relationship to iron status parameters.

Iron deficiency decreased the mRNA expression of hepcidin in the animal studies similar to what was been observed in non-pregnant populations [37]. Rat mothers that were fed an iron deficient diet had lower hepcidin values [59]. Their hepcidin was significantly higher at 21.5 days if iron was supplemented in the second half of pregnancy, but not if supplemented in the first half [59]. Hepatic hepcidin mRNA expression was also lower in pluriparous mice compared to nulliparious mice, when hepcidin was assessed after the last delivery, suggesting that pluriparous mice had greater iron transfer to the pups [58].

### 3.3. Maternal Hepcidin and Inflammation

In general, for uncomplicated pregnancies, hepcidin levels did not correlate with inflammatory markers during gestation [49,55]. However, one study found a positive correlation between the inflammatory marker C-reactive protein (CRP) and serum hepcidin at time of delivery (gestational age 37–42 weeks) [48]. In high-risk pregnancies, such as those associated with inflammatory conditions (e.g., obesity or preeclampsia), hepcidin was elevated compared to healthy pregnancies [50,56]. In obese women during the second trimester, hepcidin was higher than in lean controls and correlated positively with maternal CRP [56]. In pregnant women with preeclampsia, plasma hepcidin, IL-6 and ferritin were all increased whereas mean corpuscular hemoglobin concentrations were decreased compared to healthy pregnant women [50]. This is not surprising given that inflammation is a known regulator of hepcidin production [71] and preeclampsia is an inflammatory state during pregnancy. Elevated maternal hepcidin during pregnancy would be expected to cause iron restriction and diminish iron availability for placental transfer. How this affects placental and fetal development is not known.

Two studies assessed hepcidin values in relationship with placental malaria [47,51]. Howard and colleges found that urinary hepcidin was positively associated with log parasitemia but not associated with hemoglobin or anemia [47]. Van Santen and colleges found no difference in hepcidin values across malaria and anemia groups [51], but this population also had no significant difference in other parameters, including serum iron, ferritin, CRP or sTfR.

### 3.4. Hepcidin and Delivery Type

Hepcidin expression was analyzed in relationship to the types of delivery and the time of sample collection in two studies [48,53]. Significantly higher hepcidin values (samples drawn < 24 h before delivery) were found in women who delivered vaginally or by emergency cesarean section compared to those who delivered by elective cesarean section [48]. Active labor is physiologically and psychologically stressful and may cause the release of cytokines such as IL-6 and stress hormones [48], that may increase hepcidin expression. This would help to explain the higher hepcidin values observed at the time of delivery for emergency cesarean section *versus* elective cesarean section, where labor had not begun. However, another study did not find differences in hepcidin between those women delivering vaginally and those undergoing an elective cesarean section when the sample was drawn before delivery [53], but in both of these groups, hepcidin values increased at three days postpartum (three-fold for vaginal delivery and five-fold for elective cesarean section) [53]. The postpartum increase in hepcidin may be related to several factors including the stress of delivery and/or the cessation of any potential suppressive signal related to the pregnancy. At three days postpartum, women with elective cesarean section had higher hepcidin than those with vaginal delivery, in contrast to the hepcidin measurements pre-delivery [48]. In this case, the recovery from surgery may be associated with more prolonged inflammation than vaginal delivery, thus stimulating hepcidin production. Abdominal surgeries such as gastrectomy, colectomy, hepatectomy, and surgery for diffuse peritonitis have been reported to be associated with increased hepcidin values during the postoperative period [72]. This could have adverse health consequences for the recovery of mothers undergoing a cesarean section.

### 3.5. Relationship between Maternal and Neonate Cord Blood Hepcidin and Iron Status

Fetal hepcidin could regulate iron transport across placenta by controlling ferroportin levels in syncytiotrophoblast. However, the relative contribution of maternal *versus* fetal hepcidin in the regulation of placental iron transport during pregnancy is unknown. Four human studies examined maternal hepcidin compared to cord blood hepcidin values drawn at delivery [48,51,52,56] and found significantly lower hepcidin in maternal serum compared to cord blood samples [48,51,52]. Lower maternal hepcidin values during pregnancy would ensure maximal iron transport to the fetus [48]. Cord blood hepcidin was associated with cord blood iron status [48], but no correlation was detected between maternal and cord blood hepcidin in any of the studies. However, in two studies, maternal hepcidin correlated with newborn cord blood iron status parameters [52]. In obese women, maternal hepcidin correlated negatively with newborn cord blood serum iron and transferrin saturation [56]. In a study examining maternal absorption and placental transfer of isotopically-labeled non-heme and heme iron, the transfer of iron to the fetus inversely correlated with maternal hepcidin (non-heme ^57^Fe *p* = 0.002, *r*^2^ = 0.43; heme 58Fe *p* = 0.004, *r*^2^ = 0.39) and was directly associated with neonatal hemoglobin (*p* = 0.004, *r*^2^ = 0.39; *p* = 0.008, *r*^2^ = 0.35) [52]. These findings indicate that maternal hepcidin at least in part determines fetal iron homeostasis.

In animal studies, fetal hepcidin expression correlated with their iron status. In rat studies [59], fetal hepcidin at 21.5 days was the lowest when mothers where fed an iron deficient diet throughout the pregnancy, moderate correction of hepcidin levels was observed if iron was supplemented in the first half of pregnancy, and complete correction of fetal hepcidin levels were observed if iron was supplemented in the second half of pregnancy [59].

## 4. Limitations

There are limitations to the review that warrant discussion. The small number of research studies that assess hepcidin during pregnancy limits the strength of the conclusions. The sample sizes of most individual studies were small and future research should assess hepcidin in large population studies of pregnant women. While the hepcidin measurements obtained by different methods such as ELISA and mass spectroscopy correlate well [73], the absolute hepcidin values are substantially different depending on the method and cannot be related to one another, limiting comparisons across studies. Harmonization of different hepcidin methods is needed to allow establishment of a consistent reference range across different stages of pregnancy.

The cross-sectional study design of most human studies limited our ability to draw conclusions about changes in hepcidin values throughout the pregnancy. Furthermore, very little is known about hepcidin in complicated pregnancies. Only one study examined hepcidin values in patients diagnosed with preeclampsia [50], one in obese *versus* lean women [56], and two studies examined hepcidin values in patients infected with malaria [47,51]. Future research should examine hepcidin values in relationship to other pregnancy complications, such as preterm birth and low birth weight. Increase in blood volume in pregnancy could in itself cause the decrease in hepcidin concentrations in blood. For example, albumin concentration decreases to about 80% of the non-pregnant levels [74]. However, hepcidin decrease in the third trimester is much greater suggesting that hepcidin decrease cannot be explained solely by changes in blood volume.

## 5. Conclusions

Hepcidin is a regulator of iron homeostasis and may be a useful biomarker to determine iron bioavailability in pregnancy. Proper diagnosis of true iron deficiency anemia *versus* inflammation-mediated iron restriction during pregnancy may help clinicians prescribe iron therapy appropriately. Treating the underlying disease and/or use of erythropoietic agents may be an appropriate alternative [70]. Future research is needed to examine the association between hepcidin values in pregnancy and pregnancy complications. Research examining early pregnancy hepcidin values is needed as elevated or very low hepcidin values could be used as an early diagnostic indicator of maternal iron bioavailability.

Both maternal and fetal hepcidin may determine the degree of placental iron transfer. Fetal-derived hepcidin may play a role in the regulation of Fpn expressed at the basolateral side of the syncytiotrophoblast [31] and determine the rate of iron entry into fetal circulation [35]. In fact, transgenic mice engineered to overexpress hepcidin during embryonic development spontaneously aborted *in utero* due to severe iron deficiency [57]. Maternal hepcidin, however, would regulate Fe-Tf concentrations in maternal circulation by dictating the flux of iron coming from the diet as well as storage sites, and this would determine the amount of iron presented to the placenta for uptake. Throughout pregnancy, accommodation for increased iron needs for both the mother and fetus are supported by a substantial increase in maternal dietary iron absorption, and increased iron flux to the fetus via the Fpn-rich placenta. One would expect markedly low maternal hepcidin concentrations to accommodate this transfer [5,75]. In conditions which infection or inflammation is present, maternal iron bioavailability could be significantly reduced limiting the amount of iron presented for uptake by the placenta and for transfer to the fetus. Future studies should examine the direct and indirect effects of maternal and fetal hepcidin on placental expression of iron management proteins. It remains to be determined if elevated hepcidin during pregnancy is a pathogenic factor adversely impacting maternal and fetal outcomes.

## References

[1] Andrews N.C. (1999). Disorders of iron metabolism. N. Engl. J. Med..

[2] National Research Council (2001). Dietary Reference Intakes for Vitamin A, Vitamin K, Arsenic, Boron, Chromium, Copper, Iodine, Iron, Manganese, Molybdenum, Nickel, Silicon, Vanadium, and Zinc.

[3] Greer J.P., Ovid Technologies Inc. (2009). Wintrobe’s Clinical Hematology.

[4] Corbett J.V. (1995). Accidental poisoning with iron supplements. MCN Am. J. Matern. Child Nurs..

[5] Bothwell T.H. (2000). Iron requirements in pregnancy and strategies to meet them. Am. J. Clin. Nutr..

[6] Allen L.H. (2001). Biological mechanisms that might underlie iron’s effects on fetal growth and preterm birth. J. Nutr..

[7] Scott D.E. (1972). Anemia in pregnancy. Obstet. Gynecol. Annu..

[8] Tapiero H., Gate L., Tew K.D. (2001). Iron: Deficiencies and requirements. Biomed. Pharmacother..

[9] Centers for Disease Control and Prevention (1998). Recommendations to prevent and control iron deficiency in the United States. MMWR Recomm. Rep..

[10] Reshetnikova O.S., Burton G.J., Teleshova O.V. (1995). Placental histomorphometry and morphometric diffusing capacity of the villous membrane in pregnancies complicated by maternal iron-deficiency anemia. Am. J. Obstet. Gynecol..

[11] Kadyrov M., Kosanke G., Kingdom J., Kaufmann P. (1998). Increased fetoplacental angiogenesis during first trimester in anaemic women. Lancet.

[12] Konijn A.M. (1994). Iron metabolism in inflammation. Baillieres Clin. Haematol..

[13] Godfrey K.M., Redman C.W., Barker D.J., Osmond C. (1991). The effect of maternal anaemia and iron deficiency on the ratio of fetal weight to placental weight. Br. J. Obstet. Gynecol..

[14] Collins J.F., Wessling-Resnick M., Knutson M.D. (2008). Hepcidin regulation of iron transport. J. Nutr..

[15] Cogswell M.E., Parvanta I., Ickes L., Yip R., Brittenham G.M. (2003). Iron supplementation during pregnancy, anemia, and birth weight: A randomized controlled trial. Am. J. Clin. Nutr..

[16] Blot I., Diallo D., Tchernia G. (1999). Iron deficiency in pregnancy: Effects on the newborn. Curr. Opin. Hematol..

[17] Earl R.O., Woteki C.E., Institute of Medicine (U.S.), Committee on the Prevention Detection and Management of Iron Deficiency Anemia among U.S., Children and Women of Childbearing Age, Institute of Medicine (U.S.), Food and Nutrition Board (1993). Iron Deficiency Anemia: Recommended Guidelines for the Prevention, Detection, and Management among U.S. Children and Women of Childbearing Age.

[18] Allen L.H. (1997). Pregnancy and iron deficiency: Unresolved issues. Nutr. Rev..

[19] Malhotra M., Sharma J.B., Batra S., Sharma S., Murthy N.S., Arora R. (2002). Maternal and perinatal outcome in varying degrees of anemia. Int. J. Gynaecol. Obstet..

[20] Allen L.H. (2000). Anemia and iron deficiency: Effects on pregnancy outcome. Am. J. Clin. Nutr..

[21] Gulmezoglu A.M., Mahomed K., Hofmeyr G.J., Nikodem V.C., Kramer T. (1996). Fetal and maternal catecholamine levels at delivery. J. Perinat. Med..

[22] Campos M.S., Barrionuevo M., Alferez M.J., Gomez-Ayala A.E., Rodriguez-Matas M.C., Lopez Aliaga I., Lisbona F. (1998). Interactions among iron, calcium, phosphorus and magnesium in the nutritionally iron-deficient rat. Exp. Physiol..

[23] Coghetto Baccin A., Lauerman Lazzaretti L., Duarte Martins Brandao V., Manfredini V., Peralba M.C., Silveira Benfato M. (2009). Oxidative stress in older patients with iron deficiency anaemia. J. Nutr. Health Aging.

[24] Casanueva E., Viteri F.E. (2003). Iron and oxidative stress in pregnancy. J. Nutr..

[25] Knutson M.D., Handelman G.J., Viteri F.E. (2000). Methods for measuring ethane and pentane in expired air from rats and humans. Free Radic. Biol. Med..

[26] Walter P.B., Knutson M.D., Paler-Martinez A., Lee S., Xu Y., Viteri F.E., Ames B.N. (2002). Iron deficiency and iron excess damage mitochondria and mitochondrial DNA in rats. Proc. Natl. Acad. Sci. USA.

[27] Bhaskaram P. (2001). Immunobiology of mild micronutrient deficiencies. Br. J. Nutr..

[28] McArdle H.J., Lang C., Hayes H., Gambling L. (2011). Role of the placenta in regulation of fetal iron status. Nutr. Rev..

[29] Paterson S., Armstrong N.J., Iacopetta B.J., McArdle H.J., Morgan E.H. (1984). Intravesicular pH and iron uptake by immature erythroid cells. J. Cell. Physiol..

[30] Harris E.D. (1992). New insights into placental iron transport. Nutr. Rev..

[31] Donovan A., Lima C.A., Pinkus J.L., Pinkus G.S., Zon L.I., Robine S., Andrews N.C. (2005). The iron exporter ferroportin/Slc40a1 is essential for iron homeostasis. Cell Metab..

[32] Chen H., Attieh Z.K., Syed B.A., Kuo Y.M., Stevens V., Fuqua B.K., Andersen H.S., Naylor C.E., Evans R.W., Gambling L. (2010). Identification of zyklopen, a new member of the vertebrate multicopper ferroxidase family, and characterization in rodents and human cells. J. Nutr..

[33] Ganz T. (2005). Hepcidin—A regulator of intestinal iron absorption and iron recycling by macrophages. Best Pract. Res. Clin. Haematol..

[34] Ganz T. (2006). Hepcidin and its role in regulating systemic iron metabolism. Hematol. Am. Soc. Hematol. Educ. Progr..

[35] Nemeth E., Ganz T. (2006). Regulation of iron metabolism by hepcidin. Annu. Rev. Nutr..

[36] Murphy A.T., Witcher D.R., Luan P., Wroblewski V.J. (2007). Quantitation of hepcidin from human and mouse serum using liquid chromatography tandem mass spectrometry. Blood.

[37] Ganz T. (2003). Hepcidin, a key regulator of iron metabolism and mediator of anemia of inflammation. Blood.

[38] Darshan D., Anderson G.J. (2009). Interacting signals in the control of hepcidin expression. Biometals.

[39] Hamlin F., Latunde-Dada G.O. (2011). Iron bioavailibity from a tropical leafy vegetable in anaemic mice. Nutr. Metab..

[40] Goodnough L.T., Nemeth E., Ganz T. (2010). Detection, evaluation, and management of iron-restricted erythropoiesis. Blood.

[41] Gagliardo B., Kubat N., Faye A., Jaouen M., Durel B., Deschemin J.C., Canonne-Hergaux F., Sari M.A., Vaulont S. (2009). Pro-hepcidin is unable to degrade the iron exporter ferroportin unless maturated by a furin-dependent process. J. Hepatol..

[42] Sasu B.J., Li H., Rose M.J., Arvedson T.L., Doellgast G., Molineux G. (2010). Serum hepcidin but not prohepcidin may be an effective marker for anemia of inflammation (AI). Blood Cells Mol. Dis..

[43] Roe M.A., Spinks C., Heath A.L., Harvey L.J., Foxall R., Wimperis J., Wolf C., Fairweather-Tait S.J. (2007). Serum prohepcidin concentration: No association with iron absorption in healthy men; and no relationship with iron status in men carrying HFE mutations, hereditary haemochromatosis patients undergoing phlebotomy treatment, or pregnant women. Br. J. Nutr..

[44] Briana D.D., Boutsikou T., Baka S., Boutsikou M., Stamati L., Hassiakos D., Gourgiotis D., Malamitsi-Puchner A. (2012). Perinatal role of hepcidin and iron homeostasis in full-term intrauterine growth-restricted infants. Eur. J. Haematol..

[45] Simavli S., Derbent A.U., Uysal S., Turhan N.O. (2014). Hepcidin, iron status, and inflammation variables among healthy pregnant women in the turkish population. J. Matern. Fetal Neonatal Med..

[46] Itkonen O., Parkkinen J., Stenman U.H., Hamalainen E. (2012). Preanalytical factors and reference intervals for serum hepcidin LC-MS/MS method. Clin. Chim. Acta.

[47] Howard C.T., McKakpo U.S., Quakyi I.A., Bosompem K.M., Addison E.A., Sun K., Sullivan D., Semba R.D. (2007). Relationship of hepcidin with parasitemia and anemia among patients with uncomplicated plasmodium falciparum malaria in ghana. Am. J. Trop. Med. Hyg..

[48] Rehu M., Punnonen K., Ostland V., Heinonen S., Westerman M., Pulkki K., Sankilampi U. (2010). Maternal serum hepcidin is low at term and independent of cord blood iron status. Eur. J. Haematol..

[49] Schulze K.J., Christian P., Ruczinski I., Ray A.L., Nath A., Wu L.S., Semba R.D. (2008). Hepcidin and iron status among pregnant women in bangladesh. Asia Pac. J. Clin. Nutr..

[50] Toldi G., Stenczer B., Molvarec A., Takats Z., Beko G., Rigo J., Vasarhelyi B. (2010). Hepcidin concentrations and iron homeostasis in preeclampsia. Clin. Chem. Lab. Med..

[51] Van Santen S., de Mast Q., Luty A.J., Wiegerinck E.T., van der Ven A.J., Swinkels D.W. (2011). Iron homeostasis in mother and child during placental malaria infection. Am. J. Trop. Med. Hyg..

[52] Merhi Z.O., Seifer D.B., Weedon J., Adeyemi O., Holman S., Anastos K., Golub E.T., Young M., Karim R., Greenblatt R. (2012). Circulating vitamin D correlates with serum antimullerian hormone levels in late-reproductive-aged women: Women’s interagency HIV study. Fertil. Steril..

[53] Gyarmati B., Szabo E., Szalay B., Czuczy N., Toldi G., Cseh A., Vasarhelyi B., Takats Z. (2011). Serum maternal hepcidin levels 3 days after delivery are higher compared to those measured at parturition. J. Obstet. Gynaecol. Res..

[54] Finkenstedt A., Widschwendter A., Brasse-Lagnel C.G., Theurl I., Hubalek M., Dieplinger H., Tselepis C., Ward D.G., Vogel W., Zoller H. (2012). Hepcidin is correlated to soluble hemojuvelin but not to increased GDF15 during pregnancy. Blood Cells Mol. Dis..

[55] Van Santen S., Kroot J.J., Zijderveld G., Wiegerinck E.T., Spaanderman M.E., Swinkels D.W. (2013). The iron regulatory hormone hepcidin is decreased in pregnancy: A prospective longitudinal study. Clin. Chem. Lab. Med..

[56] Dao M.C., Sen S., Iyer C., Klebenov D., Meydani S.N. (2013). Obesity during pregnancy and fetal iron status: Is hepcidin the link?. J. Perinatol..

[57] Nicolas G., Bennoun M., Porteu A., Mativet S., Beaumont C., Grandchamp B., Sirito M., Sawadogo M., Kahn A., Vaulont S. (2002). Severe iron deficiency anemia in transgenic mice expressing liver hepcidin. Proc. Natl. Acad. Sci. USA.

[58] Neves J.V., Olsson I.A., Porto G., Rodrigues P.N. (2010). Hemochromatosis and pregnancy: Iron stores in the Hfe−/− mouse are not reduced by multiple pregnancies. Am. J. Physiol. Gastrointest. Liver Physiol..

[59] Gambling L., Czopek A., Andersen H.S., Holtrop G., Srai S.K., Krejpcio Z., McArdle H.J. (2009). Fetal iron status regulates maternal iron metabolism during pregnancy in the rat. Am. J. Physiol. Regul. Integr. Comp. Physiol..

[60] Millard K.N., Frazer D.M., Wilkins S.J., Anderson G.J. (2004). Changes in the expression of intestinal iron transport and hepatic regulatory molecules explain the enhanced iron absorption associated with pregnancy in the rat. Gut.

[61] Cornock R., Gambling L., Langley-Evans S.C., McArdle H.J., McMullen S. (2013). The effect of feeding a low iron diet prior to and during gestation on fetal and maternal iron homeostasis in two strains of rat. Reprod. Biol. Endocrinol..

[62] Sozo F., Dick A.M., Bensley J.G., Kenna K., Brien J.F., Harding R., de Matteo R. (2013). Alcohol exposure during late ovine gestation alters fetal liver iron homeostasis without apparent dysmorphology. Am. J. Physiol. Regul. Integr. Comp. Physiol..

[63] Ward D.G., Roberts K., Stonelake P., Goon P., Zampronio C.G., Martin A., Johnson P.J., Iqbal T., Tselepis C. (2008). SELDI-TOF-MS determination of hepcidin in clinical samples using stable isotope labelled hepcidin as an internal standard. Proteome Sci..

[64] Kroot J.J., Laarakkers C.M., Geurts-Moespot A.J., Grebenchtchikov N., Pickkers P., van Ede A.E., Peters H.P., van Dongen-Lases E., Wetzels J.F., Sweep F.C. (2010). Immunochemical and mass-spectrometry-based serum hepcidin assays for iron metabolism disorders. Clin. Chem..

[65] Kroot J.J., Hendriks J.C., Laarakkers C.M., Klaver S.M., Kemna E.H., Tjalsma H., Swinkels D.W. (2009). (Pre)analytical imprecision, between-subject variability, and daily variations in serum and urine hepcidin: Implications for clinical studies. Anal. Biochem..

[66] Ganz T., Olbina G., Girelli D., Nemeth E., Westerman M. (2008). Immunoassay for human serum hepcidin. Blood.

[67] Kemna E.H., Tjalsma H., Podust V.N., Swinkels D.W. (2007). Mass spectrometry-based hepcidin measurements in serum and urine: Analytical aspects and clinical implications. Clin. Chem..

[68] Finkenstedt A., Bianchi P., Theurl I., Vogel W., Witcher D.R., Wroblewski V.J., Murphy A.T., Zanella A., Zoller H. (2009). Regulation of iron metabolism through GDF15 and hepcidin in pyruvate kinase deficiency. Br. J. Haematol..

[69] Blackburn S.T. (2003). Maternal, Fetal & Neonatal Physiology: A Clinical Perspective.

[70] Weiss G., Goodnough L.T. (2005). Anemia of chronic disease. N. Engl. J. Med..

[71] Ganz T., Nemeth E. (2009). Iron sequestration and anemia of inflammation. Semin. Hematol..

[72] Park K.H., Sawada T., Kosuge T., Kita J., Shimoda M., Tomosugi N., Kubota K. (2012). Surgical inflammation induces hepcidin production after abdominal surgery. World J. Surg..

[73] Kroot J.J., van Herwaarden A.E., Tjalsma H., Jansen R.T., Hendriks J.C., Swinkels D.W. (2012). Second round robin for plasma hepcidin methods: First steps toward harmonization. Am. J. Hematol..

[74] Whittaker P.G., Lind T. (1993). The intravascular mass of albumin during human pregnancy: A serial study in normal and diabetic women. Br. J. Obstet. Gynaecol..

[75] McArdle H.J., Andersen H.S., Jones H., Gambling L. (2008). Copper and iron transport across the placenta: Regulation and interactions. J. Neuroendocrinol..

